# Rationalizations and identity conflict following smoking relapse: a thematic analysis

**DOI:** 10.3109/14659891.2016.1143045

**Published:** 2016-06-01

**Authors:** Lei Hum Wee, Azlyn Azmainie Binti Ithnin, Robert West, Nihayah Mohammad, Caryn Mei Hsien Chan, Siti Saadiah Hasan Nudin

**Affiliations:** ^a^ Faculty of Health Sciences, Universiti Kebangsaan Malaysia, Kuala Lumpur, Malaysia; ^b^ Cancer Research Institute, Ministry of Health, Malaysia; ^c^ Cancer Research UK, University College London, London, UK; ^d^ Health Behavioural Research Institute, Ministry of Health, Malaysia

**Keywords:** Motivation to quit, qualitative, smoking relapse

## Abstract

*Introduction*: Little is known about how smokers respond cognitively and emotionally to the experience of “late” relapse after the acute withdrawal phase. This study assessed the kinds of thoughts and feelings that emerge in order to provide a basis for quantitative research assessing prevalence of different types of response and implications for future quit attempts.

*Methods*: Face-to-face in-depth interviews were conducted among 14 people attending a quit smoking clinic in Malaysia who had relapsed after at least 6 weeks of abstinence. Transcripts were analyzed using thematic analysis to enable emergence of important aspects of the experience.

*Results*: Following relapse, smokers often engaged in rationalizations and activities to minimize worry about the harmful effects of smoking by switching to a lower-tar cigarette, reducing the number of cigarette smoked, attempting to reduce cigarette smoke inhalation, comparing themselves with other smokers, and minimizing the health risks associated with smoking. In some cases, smokers retained a “non-smoker” identity despite having relapsed.

*Conclusion*: Smoking relapsers rationalize their failure to quit and minimize their health risk in order to protect their image as non-smokers while it remains a source of identity conflict.

## Introduction

Most attempts to stop smoking result in relapse (Bold et al., 2015; Borland et al., 2010; Hughes et al., 2013; Hughes et al., 2014). Even after the acute withdrawal phase lasting around 4 weeks, relapse rates are high. It is possible that the way people react cognitively and emotionally to this “late” relapse could influence future quitting. Little research has been undertaken on late relapse, but it is important to inform the development of effective interventions (Hawkin et al., 2010; Krall et al., 2002; Wetter et al., 2004). To gain an improved understanding, it may be helpful to explore the thoughts and feelings experienced by smokers following relapse.

Studies have found that 75% of smokers who achieve 4 weeks of abstinence relapse within the first year (Ferguson et al., 2005; West et al., 2013), and of these a further 30% relapse after that (Wee et al., 2013). The processes that underlie behavior change (quitting—current to ex and lapsing—ex to current) is little understood (West, 2009). The transition from lapse to relapse (an initial violation of the no-smoking rule to abandonment of the quit attempt) has been found to follow a variety of trajectories, from relatively rapid re-establishing of smoking to a process of continued struggle that takes place over weeks or even months (Wee et al., 2013; West, 2009).

Marlatt’s relapse prevention theory proposes that an important factor driving this transition is the “abstinence violation effect,” in which smokers feel disappointed, lose self-confidence, and consider that having broken the rule once they might as well rescind it (Marlatt & George, 1984). One also cannot discount the underpinning influence of core cultural values on smokers’ lapses and relapses (Chassin et al., 2002; Gibbons et al., 1997; Lee et al., 2009; Nichter et al., 2003; Piasecki et al., 2006; Unger et al., 2003). In addition, smoking studies outside of the West are rare (Abdullah et al., 2004; Zhu et al., 2010).

It is worth taking into account the idiomatic Asian concern of “saving face” in this setting (Qi, 2011). This concept has been explored in areas such as disability and breaking bad news (Keil et al., 2007). It is an area that has been rarely explored within the context of smoking relapse. It is possible that smokers might seek to defend their positive self-esteem or sense of invulnerability by minimizing the significance of a lapse. Without an open-ended assessment of the thoughts and feelings of smokers following the relapse, it is not possible to know whether either or both of these kinds of reaction are present, or indeed others that have not been previously examined.

Therefore, this study sets out to explore the thoughts and feelings that characterize the response to the experience of “late” relapse following a smoking cessation attempt after the initial withdrawal phase.

## Method

### Sampling and recruitment

Smokers from the Quit Smoking Clinic at Hospital Tengku Ampuan Rahimah, Malaysia, were recruited for the study. This study relied on purposive sampling strategies where participants were selected to represent the diverse range of characteristics of attendees from this clinic by age, education, and number of cigarettes smoked daily.

All participants, who had relapsed after at least 6 weeks of having successfully quit smoking, were over the age of 18 years and were willing to take part in the interview. Each participant was given the chance to ask questions and receive clarification with regard to their involvement in the study. Written informed consent was obtained in accordance with medical research ethics committee approval. Confidentiality was assured by assigning pseudonyms to participants and removing all identifying information from transcripts.

The study was conducted over an 11-month period from March 2013 to February 2014. Participants underwent face-to-face, one-to-one in-depth interviews until data saturation (assessed as no new relevant information) was achieved by the eleventh participant (Silverman, 2004). This was done by two researchers (LHW and AABI), who were involved in conducting each interview as well as in the review of the recordings and agreement upon data saturation. Additional checks to verify data saturation were conducted with two further interviews.

The researcher selected a quiet room within the quit smoking clinic to ensure the highest quality of the recording and to protect the confidentially of the participants (Bryman, 2012). Interviews were guided by an interview schedule. Participant responses were probed for clarification. Each interview lasted for about 45 minutes to an hour. Interviews were recorded and transcribed verbatim (Kvale, 1996).

### The development of interview schedule

The face-to-face interviews used a schedule based on one that had been used in a previous study of smokers in England (Vangeli et al., 2013). The interview schedule consists of five parts: (1) smoking initiation, (2) first experience in smoking, (3) experience of quitting without clinic help, (4) experience of quitting with help, and (5) relapse following at least 6 weeks of smoking abstinence. This paper reports the themes emerging from topic 5 (Table 1).

**Table 1.** Summary of interview schedule.

1.	How old were you when you started smoking?
2.	Tell me about the first time that you smoked a cigarette.
3.	What experiences of giving up smoking did you have before quitting with the clinic?
4.	Thinking about when you quitted with the clinic, tell me about what it was like for you.
5.	What did you find most difficult about giving up smoking?
6.	Were there any aspects of giving up smoking that you found to be easier than you had expected?
7.	Did you feel any difference when you had stopped smoking?
8.	What was your experience of urges to smoke?
9.	Did you smoke at all between the time you quit and before returning back to smoking?
10.	Tell me about how you returned to smoking?
11.	How do you feel now compared to when you were not smoking?

## Analyses

The interviews were carried out in Bahasa Malaysia, the national language of Malaysia. The transcripts were translated into English and analyzed using N-VIVO 10 software. Participants’ socio-demographic data and smoking behavior were recorded.

Researchers sorted, organized, and analyzed the data thematically (Neale & Allen, 2005; Rivas, 2012).

### Coding

Data were coded by marking sections of the data and by giving them labels or names (Charmaz, 2011). In carrying out coding, the researchers named chunks of data with labels that simultaneously categorized, summarized, and accounted for each piece of data (Holloway & Wheeler, 2010). The researchers delved into the transcribed data and looked for meanings while naming the codes. The codes were named using descriptors that best fit the data. The researchers used short names for the codes (Charmaz, 2011).

### Thematic analysis

Two main steps were used for the thematic analysis. Two researchers read through each transcript twice to gain an overview of the interview data. Then, as part of a collective set, the researchers examined the transcript again to interpret what was said by the participants as a group (Minichielo et al., 2008). After reading the transcript for the third time, ideal elements were identified using an inductive approach in the formation of preliminary themes (Whittemore, 2001).

### Pilot study

Pilot interviews were conducted in Hospital Tengku Ampuan Rahimah to refine the questions. It also provided insights into additional questions (Patton, 2002).

### Quality control

The analysis process involved a process of additional review of the transcripts by a nursing sister working at a smokers’ clinic. Data were independently analyzed by the nursing sister and the researcher to guard the emerging themes against potential researcher bias and provide additional insights into themes (Barbour, 2001; Silverman, 2004). The transcripts were further reviewed and discussed with another professional expert. The researcher identified probable themes on the basis of the quotations. A preliminary outline was developed with similar concepts clustered together (Neuman, 2011). Participants were then shown the resulting themes and asked to check whether these reflected their experiences. All participants agreed to the key points presented to them, and only minor comments were made to suggest changes.

## Results

### Participant characteristics

Thirteen male participants were recruited with an age range of 20 to 58 years (35 ± 10.93 years old). The majority of them had completed their secondary education, and only one was educated till primary school level. Most were married (*n* = 9) (Table 2).

**Table 2.** Participants’ characteristics and smoking habits.

Name	Age (yrs)	Education level	Marital status	Occupation	No. of smokers/household	Age started smoking (yrs)	No. of sticks smoked per day	FTND	CO ppm	Cessation aid used	Longest time quit
Zaidi	22	Diploma	Single	Clerk	3	15	10	5	8	Gum	3 mths
Nawi	26	Diploma	Single	IT technical	2	18	7	4	7	Gum	2 mths
Ridhuan	27	Diploma	Single	Security Officer	1	15	10	6	9	Gum	1 year
Habil	29	SPM	Married	Factory worker	3	16	3	4	3	Gum	6 mths
Baslan	42	SPM	Married	Web designer	1	20	10	6	11	Gum	1 ½ mths
Amran	20	SPM	Single	Technician	4	14	5	2	8	Gum	1 ½ mths
Faizal	31	Diploma	Married	Clerk	3	15	7	5	9	Gum and patch	1 ½ mths
Manrizal	33	SPM	Married	Security officer	1	12	3	5	5	Lozenges	6 mths
Quay	49	SPM	Married	Property agent	1	13	20	7	14	Lozenges	1 ½ mths
Abu Badar	58	SPM	Married	Security officer	1	15	60	8	11	Gum	2 ½ yrs
Ramu	46	SRP	Married	Metal collector	1	18	20 (Mix Commercial + Roll Cig)	7	11	Gum and lozenges	1 year
Rusdan	35	SPM	Married	Medical assistant	1	14	14	5	7	Lozenges	2 ½ yrs
Said	43	SRP	Married	Star Cruise store keeper	1	13	20 (Mix Commercial + Roll Cig)	8	11	Gum and patch	8 mths

Note: mths = months; yrs = years; Cig = cigarettes; SRP = Sijil Rendah Pelajaran (lower secondary school certificate); SPM = Sijil Pelajaran Malaysia (upper secondary school certificate equivalent to O levels); FTND = Fagerstrom Test Nicotine Dependent; CO ppm = Carbon monoxide parts per million.

### List of themes

The two main themes were recurrent in at least 11 of the participant accounts. Lists of sub-themes, which emerged, are listed under the main themes (Table 3).

**Table 3.** List of main themes and sub-themes.

Main themes	Sub-themes
Rationalization of ways to reduce the harmful effects of smoking	Switching to a lower-tar cigarette
	Reducing the number of cigarettes smoked
	Comparing themselves with other smokers
	Denying the health risk associated with smoking
	Controlling cigarette smoke inhalation
Identity conflict	-

#### Rationalizations of ways to reduce the harmful effects of smoking

Smokers’ appeared to reduce the dissonance associated with resumption of smoking by (a) noting that they had switched to a “lower-tar” cigarette, (b) noting that they had reduced the number of cigarettes smoked, (c) comparing themselves with heavier smokers, (d) minimizing the health risks associated with smoking, and (e) noting that they were reducing cigarette smoke inhalation.

- Switching to a lower-tar cigarette

Said and Ridhuan tried to minimize the perceived harm of smoking by switching to lower-tar cigarettes.

… I tried cutting down on smoking, I changed it to a cool flavor, which was light [lower tar] to reduce the desire to smoke.

Said, 43 years old, CO ppm = 11, Longest time quit = 8 months

When I started smoking again, I … it’s not too heavy like how it was when I was in secondary school la. Like for now I can still control myself, meaning that like if there’s a pack I can stretch it over a day, two and a half days, I try to like reduce it la, but maybe also because I have switched from the regular brand of cigarettes I used to smoke before this.

Ridhuan, 27 years old, CO ppm = 9, Longest time quit = 1 year

- Reducing the number of cigarettes smoked

Quay and Habil tried to cut down the number of cigarettes they smoked, as explained as follows:

I try to reduce the volume, because for me next time I will quit totally much easier. I do smoke now, but much less. For example I smoke a stick [of cigarette], this 2, 3 days not really that heavy, have reduce the quantity, have reduced it.

Quay, 49 years old, CO ppm = 14, Longest time quit = 1½ months

I have now decided to make a decision to be a smoker but not a heavy smoker like before, maybe not to the point of an entire pack, but I have reduced to the point where I am probably a light smoker … within 3, 4 sticks [of cigarettes] only.

Habil, 29 years old, CO ppm = 3, Longest time quit = 6 months

- Comparing with other smokers

Some participants did not reduce their smoking but instead compared themselves favorably to heavier smokers, as seen in the following account:

I am not such a heavy smoker. If you want to consider heavy … my brother, my father, I can still be considered in control … they can finish a pack a day. I don’t smoke that much …

Faizal, 31 years old, CO ppm = 9, Longest time quit = 1½ months

When compared to my friends, there are those who smoke a pack a day, those who smoke a pack and a half a day. I feel I’m still okay … I smoke a maximum of 20 cigarettes a day.

Nawi, 26 years old, CO ppm = 7, Longest time quit = 2 months

… when I meet my friends, they smoke cigarettes, I also feel like smoking. This all like automatic … but my friends smoke the standard cigarettes, I only smoke the rolled cigarettes.

Ramu, 46 years old, CO ppm = 11, Longest time quit = 1 year

- Minimizing the health risk associated with smoking

Other strategies observed by some participants involved a denial of the health risks of smoking. As an example, Said explained:

When we smoke, male or female, people say we can become impotent that way. But I have seen my cousin brother smoke but he has a string of children … like it’s nothing … my cousin brother is a heavy smoker … We look at other people who smoke who also have children, they aren’t even impotent …

Said, 43 years old, CO ppm = 11, Longest time quit = 8 months

- Controlling cigarette smoke inhalation

Other strategies observed included controlling the amount of cigarette smoke inhaled. As an example, Abu Badar explained that he controlled his nicotine intake to obtain the amount of nicotine he needed to sustain his addiction.

Emmm … right now, I do smoke … but I don’t fully smoke la, I breathe it in a little and then exhale … I don’t take it in fully, not inhaling it. Prior to this yes, I used to be a heavy smoker, sometimes the filter would finish to the end before I would toss it. I truly was a heavy smoker … I inhaled right into the chest. But now I don’t inhale as deeply, I smoke a little and then I just throw it away.

Abu Badar, 58 years old, CO ppm = 11, Longest time quit = 2½ years

I don’t crave nicotine, I have never been one to have a cigarette while watching television or reading the paper. No more. Now I just inhale a little not as much as before.

Quay, 49 years old, CO ppm = 14, Longest time quit = 1½ months

When I want to smoke, I smoke one puff, then I give it to my member [friend], I give it all away.

Amran, 20 years old, CO ppm = 8, Longest time quit = 1½ months

#### Identity conflict

This theme demonstrates the conflict faced by smokers in deciding to quit or remain a smoker.

Manrizal explained his conflict between choosing to remain abstinent or to continue smoking if he could merely beat his addiction to cigarettes.

I can quit smoking, but the problem is that my desires surpass my efforts. I can’t … If my intentions were to quit … at this time I am not ready yet. Actually I do want to stop smoking, but I just go with it … [laugh] … people say it’s a false front that conceals a silent desire.

Manrizal, 33 years old, CO ppm = 5, Longest time quit = 6 months

Quay explains that he doesn’t think of himself as a smoker, even though he often lights up with friends. For him, real smokers are people who can’t make it through a day without their cigarettes, Quay explains as follows:

I don’t crave nicotine, I’ve never been one to have a cigarette while watching TV or reading the paper. No more. Now I just smoke a little only not fully like before.

Quay, 49 years old, CO ppm = 14, Longest time quit = 1½ months

Zaidi is motivated to quit because of his promise to his fiancée and the state of his finances. His low motivation to quit stopped him from seeking clinical help earlier. He experienced identity conflict to seek help to quit or continue as a smoker for a long time.

I did intend to quit smoking because of my fiancée, she did request that I do so. Another matter aside from the aspect of finances, never mind money, that made me want to quit. It’s just that my enthusiasm stops me from attending the quit smoking clinic. That’s why I have felt undecided, undecided until now.

Zaidi, 22 years old, CO ppm = 8, Longest time quit = 3 months

Baslan felt that it was more normal to smoke than not to smoke, and he states that he misses his former identity as a non-smoker.

Ha, after I smoke I feel normal again after smoking … just like before how I felt before quitting smoking.

Baslan, 42 years old, CO ppm = 11, Longest time quit = 1½ months

I avoid smoking openly … Can smoke, but like people say, you must clever clever [hide] …

Rusdan, 35 years old, CO ppm = 7, Longest time quit = 2½ years

## Discussion

This study identified a wide range of rationalizations and activities that reduced dissonance at having relapsed. In Asian culture, it is important to “save face,” a concept and absolute concern that may not be fully appreciated by Westerers (Ho, 1976). According to Qi (2011), face-saving allows the attribution of blame to fall on something else other than the individual self and hence avoid embarrassment. It permits smokers to continue attending the clinic and retain the belief that they are helping themselves, in the face of conflicting evidence.

The need to save face may allow relapsers to be open about their concurrent smoking and to admit it, but not look bad. Rationalizing lapses and minimizing health risks permits smokers to maintain their self-image as non-smokers, but it remains a source of identity conflict. When such dissonance is experienced, smokers may also visit clinic to avoid situations which remind or present direct evidence of their failure to quit.

This study offers an insight into the beliefs and many ways that individuals who are trying to quit smoking may attempt to downplay the harmful effects of smoking. Findings of this study also supports the promotion of the “not a puff” rule and non-smoker identity to prevent smoking relapse, as these have been shown to be vulnerabilities among smokers (Vangeli et al., 2010).

Implications abound for clinicians involved in assisting smokers’ quit attempts. Healthcare workers and researchers in this capacity should consider core social values which inherently influence smoking cessation attempts. This is important as the majority of smokers today are from both developing and transitional nations, such as in the current study setting (Turk & El-Khoury, 2014).

As smokers often offer explanations to try face-saving (i.e., drawing comparison to heavier smokers), and rationalize and minimize their lapses, an indirect approach may be useful to help smokers develop insight and awareness of their situation (Davidescu et al., 2014). Healthcare workers should encourage smokers, at first, to express their own explanations and then subsequently help them challenge their thought processes in order to begin moving forward toward a resolution of their current smoking behavior.

While there are a number of efficacious therapies in managing nicotine addiction (Cahill et al., 2014), we need to be able to better understand the beliefs and cultural norms that underlie smokers’ thoughts and behaviors in smoking cessation process in this setting, which needs further empirical exploration.

The study had several limitations. However, it did appear to be sufficient to reach saturation in terms of themes identified. The sample size was small, and our findings may not be applicable to a population of non-treatment-seeking smokers who may hold different beliefs and motivation to quit. Nonetheless, this qualitative analysis offers much-needed insight into a rarely studied population. This research is driven by a theoretical framework, rather than phenomenological observation, and used open-ended questions which allowed deeper exploration of the themes that were of interest.

## Conclusion

Relapse to smoking following a quit attempt can lead to a wide range of activities and thoughts that minimize emotional conflict and preserve identity as a non-smoker.
